# Retracted: A Survey of Home Delivery and Newborn Care Practices among Women in a Suburban Area of Western Nigeria

**DOI:** 10.1155/2015/689596

**Published:** 2015-08-11

**Authors:** International Scholarly Research Notices

The paper titled “A Survey of Home Delivery and Newborn Care Practices among Women in a Suburban Area of Western Nigeria” [1] has been retracted as it is found to contain a substantial amount of material from the published paper in BMC Pregnancy and Childbirth 2006 titled “Home delivery and newborn care practices among urban women in western Nepal: a questionnaire survey” by Chandrashekhar T. Sreeramareddy, Hari S. Joshi, Binu V. Sreekumaran, Sabitri Giri, and Neena Chuni.
